# Quantitative Evaluation of Tissue Surface Adaption of CAD-Designed and 3D Printed Wax Pattern of Maxillary Complete Denture

**DOI:** 10.1155/2015/453968

**Published:** 2015-10-25

**Authors:** Hu Chen, Han Wang, Peijun Lv, Yong Wang, Yuchun Sun

**Affiliations:** Center of Digital Dentistry, Faculty of Prosthodontics, Peking University School and Hospital of Stomatology and National Engineering Laboratory for Digital and Material Technology of Stomatology and Research Center of Engineering and Technology for Digital Dentistry of Ministry of Health, 22 Zhongguancun Nandajie, Haidian District, Beijing 100081, China

## Abstract

*Objective*. To quantitatively evaluate the tissue surface adaption of a maxillary complete denture wax pattern produced by CAD and 3DP. *Methods*. A standard edentulous maxilla plaster cast model was used, for which a wax pattern of complete denture was designed using CAD software developed in our previous study and printed using a 3D wax printer, while another wax pattern was manufactured by the traditional manual method. The cast model and the two wax patterns were scanned in the 3D scanner as “DataModel,” “DataWaxRP,” and “DataWaxManual.” After setting each wax pattern on the plaster cast, the whole model was scanned for registration. After registration, the deviations of tissue surface between “DataModel” and “DataWaxRP” and between “DataModel” and “DataWaxManual” were measured. The data was analyzed by paired *t*-test. *Results*. For both wax patterns produced by the CAD&RP method and the manual method, scanning data of tissue surface and cast surface showed a good fit in the majority. No statistically significant (*P* > 0.05) difference was observed between the CAD&RP method and the manual method. *Conclusions*. Wax pattern of maxillary complete denture produced by the CAD&3DP method is comparable with traditional manual method in the adaption to the edentulous cast model.

## 1. Introduction

Before the complete dentures are finally produced and the problems become uncorrectable, a procedure of try-in refers to the wearing of a wax pattern, after the arrangement of artificial teeth, in the mouth of an edentulous patient to identify and fix any problems with denture design. Thus, complete denture try-in is a vital step in the design of complete dentures for restoring edentulous jaws.

Several key points should be confirmed during the try-in [1, 2], including good fit of the completed dentures on the edentulous alveolar ridge and no tilting, twisting, nor stretching of the denture base. Vertical distance, centric relation, occlusion, aesthetics, and phonetic function should also be checked. To best test these dynamic functions of complete denture requires that the dentures be of good retention and stability; that is, each denture must have a snug fit between the denture tissue surface and the edentulous alveolar ridge.

Computer-aided design (CAD) and computer-aided manufacturing (CAM) technology has been widely applied in multiple oral disciplines, especially in the field of fixed denture restoration [3–5]. However, the application of CAD/CAM in the design and development of complete dentures is still being explored. Kawahata et al. and Inokoshi et al. applied the RP technology in the coping of a complete denture [6, 7]. In their studies, a wax copy of old complete denture was produced and used to do try-ins to evaluate the aesthetics, comfort, and stability in patients. However, no studies have quantitatively evaluated the tissue surface adaption of 3D printed dentures in patients, and none have compared this method quantitatively against the traditional manual method. In the previous study we developed the first generation of complete denture CAD software using a rapid prototyping (RP) process to complete denture production [8]. Systematic study of this entire CAD/RP process, from the acquisition of data to digital design and production, has since been carried out. However, a successful digital try-in methodology for complete denture restoration has not yet been established.

In this study, a wax pattern of maxillary complete denture was made on a standard edentulous plaster cast using high-precision 3D wax-printing technology, and the fit between the wax pattern and the cast was evaluated quantitatively.

## 2. Materials and Methods

### 2.1. Complete Denture Wax Pattern CAD

**Figure 1:**
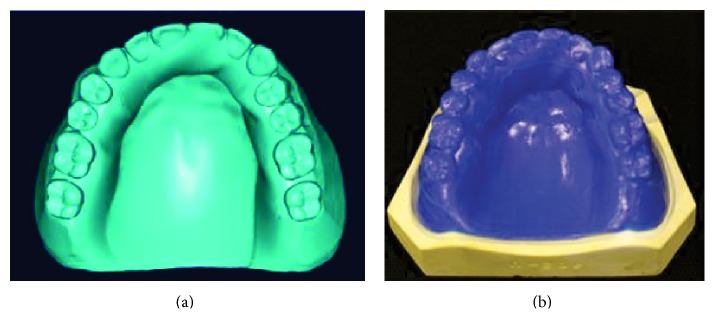
Production of the wax pattern: (a) designed in the CAD software; (b) 3D printed.

3D data of the standard edentulous maxilla plaster cast model (DataModel) and its temporal denture base were obtained using a 3D scanner (Activity 880, Smart Optics, Germany). CAD software for complete denture was developed based on a reverse engineering software (Imageware 10.0, Siemens, Germany), where the 3D data of the plaster cast and the temporal denture base were registered, and the wax pattern of complete denture was designed (Figure 1(a)).

### 2.2. Wax Denture Manufacturing

The CAD denture data were imported into the supporting software of the 3D wax printer (ProJet CPX 3500, 3D Systems, USA) where, with a print layer thickness of 20 *μ*m, the wax complete denture was printed (Figure 1(b)). A second wax denture was also created on the same plaster cast using the traditional manual method.

### 2.3. Wax Denture Scanning

The 3D printed wax pattern was scanned to obtain “DataWaxRP.” After setting the denture on the plaster cast completely, the entire model was scanned to obtain “DataModel&WaxRP.” Similarly, the wax denture made in the traditional manual method was scanned to obtain “DataWaxManual,” and the entire model, after setting the denture on the plaster cast, was scanned to obtain “DataModel&WaxManual.”

### 2.4. Data Registration

The above scan data were inputted together into a reverse engineering software (Geomagic Studio/Qualify 2013, Geomagic, USA). Using the multipoint registration and best fit alignment command, “DataWaxRP” was registered to “DataModel&WaxRP” and “DataWaxManual” to “DataModel&WaxManual,” where the polished surface of the wax denture was set as the common area. Similarly, “DataModel” was registered to “DataModel&WaxRP” and “DataModel&WaxManual” with the uncovered surface of the plaster model as the common area.

### 2.5. 3D Deviation Analysis

**Figure 2:**
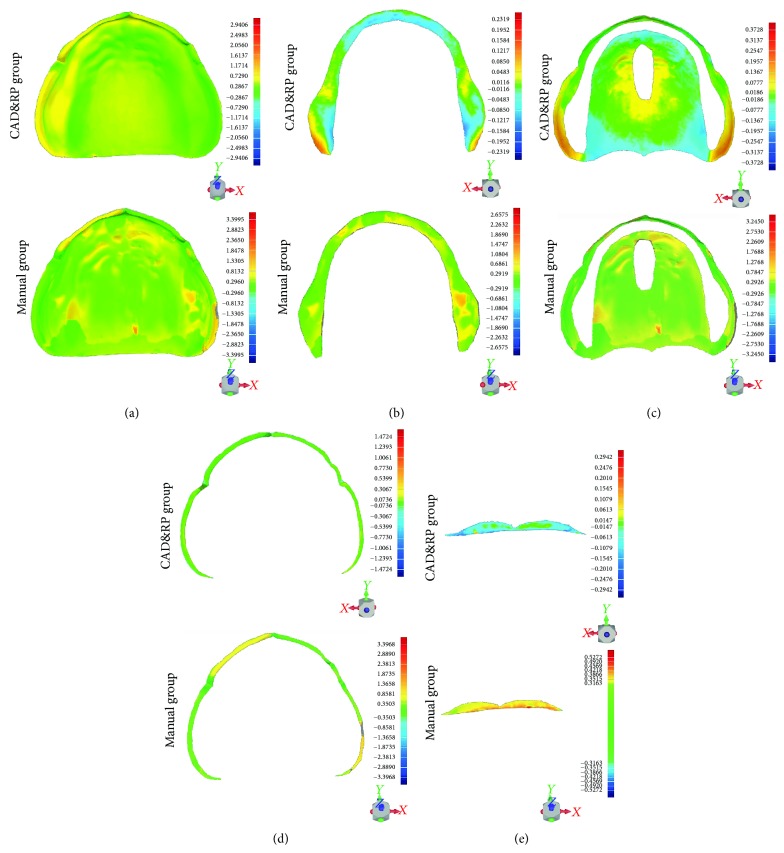
Deviation between tissue surfaces of cast model and wax pattern for CAD&RP group and manual group: (a) overall; (b) primary stress-bearing area; (c) secondary stress-bearing area; (d) border seal area; (e) postdam area.

After registration, using the 3D deviation analysis command, average deviations of the overall area, primary stress-bearing area, secondary stress-bearing area, and border seal area between “DataWaxRP” and “DataModel” (CAD&3DP group) and between “DataWaxManual” and “DataModel” (manual group) were measured (Figure 2).

### 2.6. Statistical Analysis

3D deviation analysis data of functional areas (primary stress-bearing area, secondary stress-bearing area, border seal area, and postdam area) were analyzed in statistical analysis software (SPSS 19.0, IBM, USA) using paired *t*-test where the CAD&3DP group is set as the experimental group and the traditional manual method group as the control group. *P* value above 0.05 was considered to be not statistically significant.

## 3. Results

Deviation between the tissue surface of the wax denture and the plaster cast was shown in Figure 2, the green area represents a good fit between them, yellow and red areas indicate a positive deviation (tissue surface locating above the cast surface), and the blue area indicates the presence of a negative deviation (tissue surface locating below the cast surface). For both the experimental group and the control group, scanning data of tissue surface and cast surface showed a good fit with each other in the majority, regardless of some small proportion of positive deviation area. Almost no region has shown negative deviation, since the actual surface of the denture base cannot invade the surface of the cast model. The very small range of blue region presented on the buccal side of left maxillary tuberosity in control group may be explained as a scanning error by accident. Deviation of the experimental group focused on the right maxillary alveolar crest and buccal edge region, followed by the palate region, with maximum distance below 0.7 mm. Deviation of the control group distributed scattered with distance above 1.0 mm in some regions. The average deviations between the tissue surface of the wax denture and the plaster cast were listed in Table 1. Paired samples *t*-test between functional areas of CAD&3DP group and manual group has shown no significant difference.

## 4. Discussion

3D printing or 3DP for short was developed by MIT (Massachusetts Institute of Technology) and is based on droplet ejection technology. 3DP utilizes an electromechanical integration system consisting of mechanical technology, control technique, and computer technology, while the 3D printer is made up of an ejection nozzle, mechanical system, numerical control system, and layered software. 3DP is one kind of the RP technology, which is a general term for technology driven directly by the CAD model to rapidly manufacture an arbitrary complex 3D entity. The basic process is as follows. Firstly, the 3D digital model of the required parts is designed. Usually, the digital model is sliced into several layers by a certain thickness in the *Z* direction. The original 3D model is then divided into a series of layers. Inputting the processing parameters based on the contour information of each layer sheet allows for the automatic generation of the numerical code. Finally, a 3D physical entity consisting of a series of layers is formed by stacking. This technology [9–11] can receive CAD data directly and create new samples or models quickly without need for molds, cutting tools, or tooling fixtures. From the traditional “subtractive manufacturing” to current “additive manufacturing” and from mold manufacturing to no mold manufacturing, this technology is suitable not only for the manufacturing of quick, single piece and small batch parts, complex shape products, mold, or models, but also for materials that are otherwise unmanageable and materials for exterior design inspection, assembly testing, and rapid reverse engineering.

The ProJet CPX 3500 3D wax printer can print the computer-based 3D digital model layer by layer using 3DP technology. There is a difference in the melting point between the building material and support material used (building material, VisiJet Hi-Cast, melting point 70°C; support material, VisiJet S400, melting point 55–65°C); the support material is dissolvable and allows for removal from delicate wax parts without damage to pattern surfaces or fine features. This printer can produce wax models with high precision and a delicate surface, which can be used for casting jewelry, medical equipment with microscopic detail, medical implants, and electrical components.

Since wax is thermoplastic, micro-shrinkage deformation occurs during the curing process from a liquid to a solid state. For wax pattern of complete dentures, micro-shrinkage deformation may have effect on the adaption of the tissue surface. Nevertheless, there was no statistically significant difference observed between CAD&3DP group and manual manufacturing group for measurements of deviation between the denture tissue surface and the plaster cast model. This finding demonstrated that the CAD&3DP method, using our complete denture CAD software and the ProJet CPX 3500 printer, can meet the clinically acceptable precision for design and development of complete dentures for try-in for restoring edentulous jaws.

In this study, a wax pattern of complete denture, which allows the evaluation of occlusion, vertical distance, central relationship, aesthetics, and pronunciation, was produced using the digitalized method. Importantly, modifying of the wax pattern could be done during try-in and the improved profile fed back into the design software for further manufacturing.

However, it takes a relatively long time to print the wax pattern of complete denture (14–16 hours) and removing the support material (1-2 hours). The adaption of the tissue surface of wax pattern to the cast for mandibular complete denture should also be evaluated in further study.

## 5. Conclusion

Compared with the traditional manual method, the adaption of the maxillary complete denture prepared using the CAD&3DP method for testing teeth arrangement and the edentulous alveolar ridge was not significantly different, meaning that the CAD&3DP method can meet the clinically acceptable precision for design and development of complete dentures for try-in for restoring edentulous jaws.

## Figures and Tables

**Table 1:** Average deviation between tissue surface of wax denture and plaster cast.

Area	Deviation (mm)	
	CAD&3DP group	Manual group
Overall	0.29 ± 0.14	0.30 ± 0.17
Primary stress-bearing area	0.33 ± 0.15	0.29 ± 0.18
Secondary stress-bearing area	0.27 ± 0.12	0.29 ± 0.17
Border seal area	0.41 ± 0.21	0.35 ± 0.30
Postdam area	0.29 ± 0.07	0.34 ± 0.05
